# Co-use of opioids and cannabis versus single-substance use: a national analysis of US adults

**DOI:** 10.3389/fpubh.2025.1623420

**Published:** 2025-12-08

**Authors:** Hyojung Kang, Jilin Tian, Gary Milavetz

**Affiliations:** 1 Health and Kinesiology, University of Illinois Urbana-Champaign, Champaign, IL, United States; 2 Health Care Engineering Systems Center, University of Illinois at Urbana-Champaign, Champaign, IL, United States; 3 Department of Biostatistics and Bioinformatics, Duke University, Durham, NC, United States; 4 College of Pharmacy, The University of Iowa, Iowa City, IA, United States

**Keywords:** opioids, cannabis, co-use, substance use, national analysis, NSDUH

## Abstract

**Background:**

While many studies have explored the relationship between cannabis and opioid use, few have examined how individuals who use opioids only, cannabis only, or both substances differ in terms of sociodemographic and health-related characteristics. Understanding these differences may support the development of clinical and public health strategies addressing substance use patterns.

**Methods:**

We analyzed data from the 2015–2022 National Survey on Drug Use and Health (NSDUH), focusing on US adults (≥18 years) who reported past-year medical use of prescription opioids and/or cannabis. Individuals were categorized into three mutually exclusive groups: opioid-only, cannabis-only, and opioid-cannabis co-use (OC). Descriptive statistics and weighted multinomial logistic regression models were used to compare characteristics across groups, adjusting for the complex survey design.

**Results:**

Among 134,402 adults, 49.5% used opioids only, 35.3% used cannabis only, and 15.2% reported co-use. Co-use was more common among younger adults, individuals with lower income, and those experiencing psychological distress. The impact of depression on the relative risk ratio (RRR) for opioid use only, relative to co-use, was similar to those on the RRR for cannabis use only (RRR = 0.52; 95% CI: 0.49–0.56). However, for many characteristics, the RRR of opioid use only, vs. co-use, differed from that of cannabis use only, vs. co-use. Compared to co-users, individuals in the opioid-only group were more likely to be older, women, and reside in large-metro areas. In contrast, individuals in the cannabis-only group were more likely to be younger, men, report better health status, and reside in non-metro regions.

**Conclusion:**

Individuals who use opioids only, cannabis only, or both substances differ significantly across demographic, socioeconomic, and health-related factors. These distinctions highlight the need for tailored clinical guidance and public health responses that account for co-use patterns and geographic context to support safer pain management and substance use care.

## 1 Introduction

Prescription opioids are commonly used to reduce acute and chronic pain. In the US, the overall opioid dispensing rate per 100 persons exhibited a continuous increase from 72.4 to 81.3 between 2006 and 2012 (1). However, in response to policy changes and stricter provider regulations, there was a subsequent decline in opioid prescriptions until 2020 (1). While opioids play a significant role in pain management within medical settings, their highly addictive nature carries substantial risks of misuse and overdose (2), which contributes to a broader public health crisis.

As an alternative to prescription opioids for managing pain, cannabis has increasingly been considered. Although cannabis remains a federally controlled substance in the US, as of February 2025, 39 states and the District of Columbia have legalized its use for medical purposes (3). Studies have shown that cannabis may provide benefits, particularly in the management of chronic pain (4–6). In addiction, several studies have reported that individuals with pain conditions often prefer using cannabis over opioids to manage their symptoms (7–9). While the risk of addiction is lower than that of opioids, approximately 1 in 10 people who use cannabis develop an addiction, with the risk significantly higher among those who begin use before age 18 (10). Cannabis use has also been linked to an increased risk of developing psychotic disorders, including schizophrenia (11, 12).

A growing body of research has examined the impact of increased access to cannabis on opioids use (13–15), while other studies have assessed the likelihood that individuals who are prescribed opioids will also use cannabis (16). In addition, studies have explored the risk associated with co-use of opioids and cannabis. For example, among individuals who used cannabis, the risk of developing nonmedical prescription opioid use and opioid use disorder increased (17, 18). Moreover, individuals who use both opioids and cannabis were more likely to experience higher levels of anxiety and depression and to engage in additional substances compared to those who used opioids alone (19, 20). Given these interactions and the potential for one substance to influence the use of the other substance, it is important to better understand the characteristics of individuals who use opioids only, cannabis only, and both substances. Such understanding could help identify individuals at higher risk for co-use and related harms. However, few studies have focused on the distinctive characteristics of these groups. Prior work comparing demographic characteristics of individuals who use opioids only versus use both opioids and cannabis has largely focused on older adults (21, 22), and no studies to date have directly compared individuals who use cannabis but not opioids with those who use both.

The objective of our study is to characterize and compare three groups: individuals who used opioids only (hereafter referred to as the “opioid-only” group), those who used cannabis only (“cannabis-only” group), and those who used both (“OC” group). Using multinomial logistic regression, we identify significant demographic, socioeconomic, and health status factors associated with membership in each group. Then, we discuss how the findings can inform clinical guidelines and public policies to promote safer management of opioid and cannabis use and to help prevent potential adverse outcomes associated with their concurrent use. For this study, we focused on prescription opioid use; however, the data did not differentiate between medical and recreational cannabis use. As a result, our analysis reflects self-reported cannabis use without this distinction, which should be considered when interpreting the findings.

## 2 Methods

### 2.1 Study settings

This study utilized data from the National Survey on Drug Use and Health (NSDUH), collected by the Substance Abuse and Mental Health Services Administration from 2015 to 2022 (23). The data were collected via an annual, cross-sectional survey that asks non-institutionalized individuals aged 12 years and older about their experience with substance use and health status. To provide population estimates, the survey uses a stratified, multi-stage area probability sampling design. Details of the sampling design and methods used for the survey are provided elsewhere (24). Also, the survey does not collect any identifiable information. We focused on adults (aged 18 years and older) because adolescents have different substance use behaviors and outcomes from adults (25). For example, adolescents are more prone to engage in risky substance use behaviors, which can be influenced by social and developmental factors, such as peer substance use (26, 27). This study was exempt from IRB review because the NSDUH data are de-identified and publicly available, which means it does not constitute human subject research. As a result, participant consent was not required.

### 2.2 Outcome variables

The main outcome variables of this study are prescription pain reliever (opioid) and/or cannabis use in the past year. The NSDUH survey included the following question: “In the past 12 months, which, if any, of these pain relievers have you used?” A “yes” response indicated that the individual had used a prescription opioid in the past year. We excluded individuals who reported misusing prescription opioids in terms of frequency, amount, and duration, as prior research indicates that nonmedical use is associated with distinct drug use patterns and a higher likelihood of polysubstance use, including cannabis (28–30). To ensure a more focused analysis, we limited our sample to individuals who reported medical use of opioids. Another question was, “How long has it been since you last used marijuana or hashish?” If the response was either “within the past 30 days” or “more than 30 days ago but within the past year,” we considered that as cannabis use within the past year. Using the responses to those two questions, we defined the following groups: (1) opioid-only (yes to opioid question, no to cannabis question), (2) cannabis-only (no to opioid question, yes to cannabis question), and (3) OC group (yes to both questions).

### 2.3 Independent variables

Demographic factors included age, gender, and race/ethnicity. Age was grouped into young (18–34 years old), middle-aged (35–64 years old), and older adults (65 + years old). Racial/ethnic groups were classified into non-Hispanic (NH) white people, NH black people, Hispanic people, and other minorities. Socioeconomic and geographic factors included family income (<$20,000, $20,000–$49,999, $50,000–$74,999, >$75,000), education completed (less than or higher than high school diploma/GED credential), health insurance type (private, Medicaid/Medicare, self-pay, other), employment status (unemployed, employed), and metropolitan statistical area (non-metro, small metro, large metro areas).

We considered the health status of participants. Overall self-reported health status (question: “Would you say your health in general is excellent, very good, good, fair, or poor?”) was categorized into four levels (Fair/Poor, Good, Very Good, Excellent). Mental health status was estimated using a serious psychological distress indicator based on the worst Kessler-6 total score in the past year (31). The survey year (2015–2022) was included as a covariate.

### 2.4 Statistical analysis

First, we used descriptive statistics to compare the characteristics of the opioid-only, cannabis-only, and OC groups. Continuous variables were compared using one-way ANOVA tests with the assumption of equal variances, and categorical variables were compared using chi-square tests (*α* = 0.05). Next, we used a weighted multinomial logistic regression model to evaluate factors associated with co-use, comparing the OC group to both the opioid-only and cannabis-only groups. To examine potential multicollinearity among categorical independent variables, we computed generalized variance inflation factor (GVIF), which extend the traditional VIF to handle multi-level categorical predictors (32). All adjusted GVIFs were less than 2, which indicates no significant multicollinearity. Model adequacy was assessed using a likelihood ratio test that compares the full multinomial logistic regression model to the intercept-only model. All analyses incorporated survey weights and accounted for the complex survey design of NSDUH, including clustering and stratification. Statistical analyses were conducted using the R software, version R 4.3.0 (33). Specifically, we constructed a complex survey design object using the final analysis weight (ANALWT_C), clustering (VEREP), and stratification variable (VESTR) from the NSDUH data, implemented via the *svydesign* function in *survey* package. A weighted multinomial logistic regression model was then fitted using *svy_vglm* function from *svyVGAM* package in R (32).

## 3 Results

After individuals younger than 18 years old or who did not use either opioids or cannabis in the past year were excluded, 134,402 participants were included in this study; that extrapolated to 96,651,727.50 in the weighted sample for 2015–2022. We used NSDUH data in which imputation had already been performed, and therefore no missing values remained in our analyses (32).

Of the participants, 49.51% (*n* = 66,548) used opioids only, 35.34% (*n* = 47,497) used cannabis only, and 15.15% (*n* = 20,357) used both substances in the past year. Table 1 summarizes the characteristics of the three groups based on opioid and/or cannabis use status in the past year: opioid-only use, cannabis-only use, and OC co-use. More than 50% of the opioid-only group were middle-aged adults (53.4%), and 27.3% were older adults. On the other hand, cannabis-only use was most prevalent among the young adult group at 57.5%, followed by the middle-aged group at 37.3%. The prevalence of co-use of OC was similar for young adults and middle-aged adults, but the rate was low among older adults (9%).

**Table 1:** Characteristics of three participant groups based on opioid/cannabis use status.

	Group			
	Opioid use only (*n* = 57866325.1)	Cannabis use only (*n* = 25679692.9)	^CO Co-use (*n* = 13105709.5)	*p*-value
Gender (%)				
Men	24569966.7 (42.5)	15048184.8 (58.6)	6747584.7 (51.5)	<0.05
Age (%)				
18–34	11202376.5 (19.4)	14770932.0 (57.5)	5364020.9 (40.9)	<0.05
35–64	30884524.9 (53.4)	9582896.2 (37.3)	6567514.5 (50.1)	
65 or older	15779423.7 (27.3)	1325864.7 (5.2)	1174174.1 (9.0)	
Race/ethnicity (%)				
NH^#^ white people	39058694.3 (67.5)	16438905.3 (64.0)	8694875.3 (66.3)	<0.05
NH black people	7325580.1 (12.7)	3572042.0 (13.9)	1824410.8 (13.9)	
NH other minorities	3712768.7 (6.4)	1847722.8 (7.2)	839020.0 (6.4)	
Hispanic people	7769282.1 (13.4)	3821022.8 (14.9)	1747403.4 (13.3)	
Family income (%)				
<$20,000	9532057.6 (16.5)	4951080.4 (19.3)	2897716.0 (22.1)	<0.05
$20,000–$49,999	17179541.0 (29.7)	7267798.1 (28.3)	4005446.6 (30.6)	
$50,000–$74,999	9331586.9 (16.1)	3884151.5 (15.1)	1901298.6 (14.5)	
>$75,000	21823139.6 (37.7)	9576662.9 (37.3)	4301248.3 (32.8)	
Education (%)				
Higher education*	51059010.3 (88.2)	23387715.1 (91.1)	11730248.1 (89.5)	<0.05
Insurance (%)				
Private Insurance	26409396.5 (45.6)	14609196.8 (56.9)	6190899.4 (47.2)	<0.05
Medicaid/Medicare	25326668.1 (43.8)	6341360.3 (24.7)	4864885.4 (37.1)	
Other insurance	2311552.5 (4.0)	1169336.1 (4.6)	629676.5 (4.8)	
No insurance	3818708.0 (6.6)	3559799.7 (13.9)	1420248.2 (10.8)	
Employment status (%)				
Employed	31423714.8 (54.3)	18442934.5 (71.8)	8127246.3 (62.0)	<0.05
MSA^+^ (%)				
Non-metro	29827598.1 (51.5)	15446211.6 (60.1)	7311231.0 (55.8)	<0.05
Small-metro	18635426.8 (32.2)	7574843.2 (29.5)	4046046.5 (30.9)	
Large-metro	9403300.2 (16.3)	2658638.1 (10.4)	1748431.9 (13.3)	
Overall health (%)				
Fair/Poor	12718086.3 (22.0)	2778049.9 (10.8)	2901988.9 (22.1)	<0.05
Good	19373706.4 (33.5)	7481557.7 (29.1)	4272283.6 (32.6)	
Very Good	18025834.0 (31.2)	10104174.5 (39.3)	4182354.4 (31.9)	
Excellent	7748698.4 (13.4)	5315910.8 (20.7)	1749082.5 (13.3)	
Depression (%)				
Yes	7079863.2 (12.2)	5684411.7 (22.1)	3395072.5 (25.9)	<0.05

The sample size (*n*) was extrapolated to weighted estimates matching total population counts. ^OC, Opioid and cannabis. ^#^NH, Non-Hispanic. *Higher education includes a high school diploma/GED or college degree. +MSA, Metropolitan statistical area.

The proportion of individuals with a high school diploma/GED or higher education was similar across the three groups. More than 60.1% of the cannabis-only group lived in non-metro areas, while less than 10.4% lived in large-metro areas. The household income level was lowest in the OC group. For health insurance, the proportion of Medicare/Medicaid recipients was highest in the opioid-only group (43.8%) and lowest in the cannabis-only group (24.7%). On the other hand, the rate of self-payers was highest in the cannabis-only group (13.9%) and lowest in the opioid-only group (6.6%). The rate of individuals who reported excellent health status was highest in the cannabis-only group (20.7%). The rate of depression was much higher in the OC group (25.9%) than in the other two groups.

**Figure 1:**
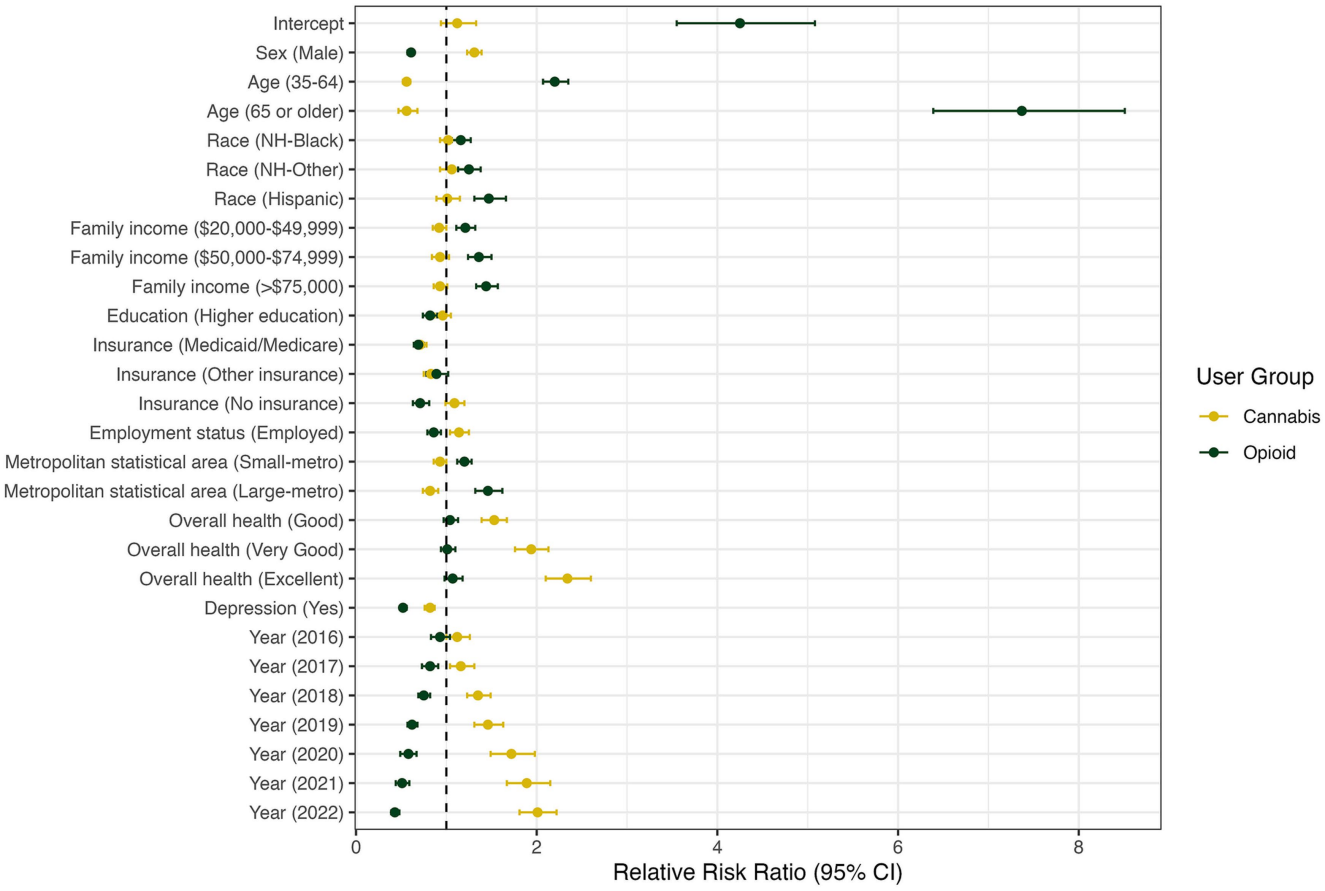
Multinomial logistic regression analysis of factors associated with opioid or/and cannabis use (baseline: co-use of opioid and cannabis).

The likelihood ratio test indicated that the full multinomial logistic regression model was statistically significant ( $\chi^{2}\;$ (54) = 30,868, *p* < 0.01). Figure 1 presents multinomial regression results that assess the relative risk ratios (RRR) for being in the opioid-only group or cannabis-only group compared to the OC group, based on characteristics. Detailed results are provided in Table 2.

**Table 2:** Multinomial logistic regression analysis of factors associated with opioid-only use or cannabis-only use (baseline: co-use of cannabis and opioid).

Variables	RRR*	95% CI	*p*-value
*Opioid-only use (baseline: co-use)*			
Gender (Women)			
Men	0.61	[0.57–0.64]	<0.001
Age (18–34)			
35–64	2.20	[2.07–2.35]	<0.001
65 or older	7.37	[6.39–8.51]	<0.001
Race/ethnicity (NH-White)			
NH-Black	1.16	[1.06–1.27]	0.001
NH-Other	1.25	[1.13–1.38]	<0.001
Hispanic	1.47	[1.31–1.66]	<0.001
Family income (<$20,000)			
$20,000–$49,999	1.21	[1.11–1.32]	<0.001
$50,000–$74,999	1.36	[1.24–1.50]	<0.001
>$75,000	1.44	[1.33–1.57]	<0.001
Education (5–12 grade completed)			
College/High school diploma/GED	0.82	[0.74–0.90]	<0.001
Insurance (Private Insurance)			
Medicaid/Medicare	0.69	[0.64–0.75]	<0.001
Other insurance	0.89	[0.78–1.02]	0.088
No insurance	0.71	[0.63–0.81]	<0.001
Employment status (Unemployed or other)			
Employed FT or PT	0.86	[0.79–0.94]	0.001
Metropolitan statistical area (Non-metro)			
Small-metro	1.20	[1.12–1.28]	<0.001
Large-metro	1.46	[1.32–1.62]	<0.001
Overall health (Fair/Poor)			
Good	1.04	[0.97–1.13]	0.272
Very Good	1.01	[0.94–1.10]	0.738
Excellent	1.07	[0.98–1.18]	0.118
Depression (No)			
Yes	0.52	[0.49–0.56]	<0.001
Year (2015)			
2016	0.93	[0.83–1.04]	0.195
2017	0.82	[0.73–0.91]	<0.001
2018	0.75	[0.69–0.82]	<0.001
2019	0.62	[0.57–0.68]	<0.001
2020	0.58	[0.49–0.67]	<0.001
2021	0.51	[0.44–0.59]	<0.001
2022	0.43	[0.39–0.48]	<0.001

Variables	RRR*	95% CI	*p*-value
*Cannabis-only use (baseline: co-use)*			
Gender (Women)			
Men	1.31	[1.23–1.39]	<0.001
Age (18–34)			
35–64	0.56	[0.52–0.60]	<0.001
65 or older	0.56	[0.47–0.68]	<0.001
Race/ethnicity (NH^#^ white people)			
NH black people	1.02	[0.93–1.12]	0.636
NH other minorities	1.06	[0.93–1.22]	0.390
Hispanic people	1.01	[0.89–1.15]	0.860
Family income (<$20,000)			
$20,000–$49,999	0.92	[0.85–1.00]	0.042
$50,000–$74,999	0.93	[0.84–1.03]	0.168
>$75,000	0.93	[0.86–1.01]	0.086
Education (5–12 grade completed)			
Higher education*	0.96	[0.87–1.05]	0.372
Insurance (Private Insurance)			
Medicaid/Medicare	0.71	[0.65–0.78]	<0.001
Other insurance	0.83	[0.75–0.92]	0.001
No insurance	1.09	[0.99–1.20]	0.067
Employment status (Unemployed or other)			
Employed	1.14	[1.04–1.25]	0.004
Metropolitan statistical area (Non-metro)			
Small-metro	0.93	[0.86–1.00]	0.063
Large-metro	0.82	[0.74–0.91]	<0.001
Overall health (Fair/Poor)			
Good	1.53	[1.39–1.67]	<0.001
Very Good	1.94	[1.76–2.13]	<0.001
Excellent	2.34	[2.10–2.60]	<0.001
Depression (No)			
Yes	0.82	[0.76–0.87]	<0.001
Year (2015)			
2016	1.12	[1.00–1.26]	0.047
2017	1.16	[1.04–1.31]	0.012
2018	1.35	[1.23–1.49]	<0.001
2019	1.46	[1.31–1.63]	<0.001
2020	1.72	[1.49–1.98]	<0.001
2021	1.89	[1.67–2.15]	<0.001
2022	2.01	[1.81–2.22]	<0.001

^#^NH, Non-Hispanic. *Higher education includes a high school diploma/GED or college degree. +MSA, Metropolitan statistical area.

For many characteristics, the relative probability of opioid use only, vs. co-use, was different from the relative probability of cannabis use only, vs. co-use. Compared to women, the relative risk that men would use opioids only, vs. both substances, was lower by a factor of 0.61 (RRR = 0.61; 95% CI: 0.57–0.64). The relative risk of using opioids only, relative to both substances, was much higher in middle-aged (RRR = 2.20; 95% CI: 2.07–2.35) and older adults (RRR = 7.37; 95% CI: 6.39–8.51) than for young adults. Also, there was a significant difference in the RRR for using opioids only versus co-use among different race/ethnicity groups. Compared to NH white people, other minority groups had a 1.16–1.47 times higher RRR for opioid use only versus co-use. The RRR for opioid use only versus co-use increased with higher household income levels, while it decreased in individuals with a higher education level. The relative risk that small-metro and large-metro residents would use opioids only, as opposed to both substances, was 1.20–1.48 times higher than that of non-metro residents. The impact of depression on the RRR for opioid use only, relative to co-use, was similar to those on the RRR for cannabis use only (RRR = 0.52; 95% CI: 0.49–0.56). However, overall health status was not significantly associated with the relative risk of using opioids only, rather than both substances.

Compared to women, the risk that men would use cannabis only, relative to both substances, was greater by a factor of 1.31 (RRR = 1.31; 95% CI: 1.23–1.39). Compared to young adults, the relative risk that middle-aged and older adults used cannabis only, relative to using both substances, was lower by a factor of 0.56 (RRR = 0.56; 95% CI: 0.52–0.60) and 0.56 (RRR = 0.56; 95% CI: 0.47–0.68), respectively. Compared to the lowest income group (<$20,000), the RRR for cannabis use only versus co-use slightly decreased in the next higher income group ($20,000–$49,999; RRR = 0.92; 95% CI:0.85–1.00). However, the difference was not statistically significant for higher income groups. The RRR for comparing cannabis use alone versus co-use was lower for individuals with Medicaid/Medicare (RRR = 0.71; 95% CI: 0.65–0.78) or “other” insurance (RRR = 0.83; 95% CI: 0.75–0.92) than for those with private insurance. For residents in large metro areas, relative to those in non-metro areas, the RRR for using cannabis only was 0.82 times lower than that of using both substances (RRR = 0.82; 95% CI: 0.74–0.91). The relative risk of co-use of the substances, as opposed to cannabis use only, was significantly higher among individuals with depression (RRR = 1.23; 95% CI: 0.75–0.89). In contrast, the relative risk of co-use compared to cannabis-only use was lower when individuals perceived their overall health as better. The RRRs for co-using the substances, versus using cannabis only, did not differ significantly across education levels and races/ethnicities.

## 4 Discussion

### 4.1 Changes in the prevalence of opioid and cannabis use, 2015–2022

Our study estimated the prevalence of independent prescription opioid use, and cannabis use, and their co-use among non-institutionalized US adults between 2015 and 2022. While describing prevalence trends can serve useful descriptive epidemiologic purposes, we did not perform formal trend analyses, as comparisons of 2020 estimates with those of prior years are discouraged because of methodological changes in the NSDUH survey implemented during the COVID-19 pandemic.

When comparing the rates between 2015 and 2022, opioid use without cannabis decreased significantly by 36.1% (from 30.2 to 19.3%), while cannabis use without opioids more than doubled (from 7.1 to 15.6%). Co-use of opioids and cannabis (OC group) increased by 30.6% (from 4.9 to 6.4%). National statistics show similar trends in opioid and cannabis use, although those figures are not conditional on other substance use. For example, between 2011 and 2020, opioid prescriptions decreased by 44% (34), and overdose deaths involving prescription opioids did not increase (35). These improvements may reflect the impact of nationwide efforts to respond to the national opioid crisis (36–38). Other studies have shown rapidly increasing cannabis use, especially among younger adults, and cannabis use disorder over the last decade (39, 40). This trend may be attributed primarily to the widespread state legalization of medical and recreational cannabis (41).

### 4.2 Prior research on opioid and cannabis use

Relatively little research has focused on distinguishing the characteristics of individuals who use opioids or cannabis only from those who use both substances, although many studies have examined the broader relationship between cannabis and opioid use. Much of existing literature has evaluated whether cannabis legalization or use influences opioid consumption, the risk of opioid use disorder, or treatment outcomes for opioid use disorder, often with mixed findings. For example, some studies have reported that opioid prescriptions, hospitalizations related to opioid misuse or dependence, and opioid overdose mortality rates were lower in states that legalized medical cannabis use (14, 42, 43). Other research indicated that greater availability of cannabis does not necessarily increase opioid use (44), and that individuals with pain may reduce or avoid opioid use by substituting medical cannabis (9, 45–47). Public health programs have also documented the use of medical cannabis as a harm reduction strategy (48–51).

### 4.3 Group differences by demographic characteristics

The main objective of this study was to examine demographic and health-related characteristics of opioid-only and cannabis-only groups in comparison with the co-use group. Interestingly, several characteristics showed distinct patterns when contrasting opioid-only with co-use versus cannabis-only with co-use.

### 4.4 Gender

For example, compared with women, men had a greater risk of using cannabis only, relative to using OC, but a lower risk of using opioids only, relative to using OC. In other words, among individuals who use cannabis, men were more likely than women to use cannabis without opioids, whereas among individuals who use prescription opioids, men were more likely than women to combine opioids with cannabis. One possible explanation for this finding is the higher prevalence of recreational cannabis use among men compared with women. Men who use cannabis for recreational purposes may be more likely to co-use alcohol more often than prescription opioids. Prior studies have shown that the simultaneous use of alcohol with cannabis is the most frequent pattern of polysubstance use among individuals who use cannabis for recreational purposes (52, 53), and this co-use pattern is more common among men than women (54).

### 4.5 Age

Similarly, different age groups showed contrasting patterns in their risks of co-use relative to single-substance use. The risk that individuals in the middle-aged and older adult groups would use OC, relative to their chance of using cannabis alone, was about 1.8 times higher than for the younger adult group. However, the two older age groups had a 2.2 to 7.37 times higher probability of using opioids only, relative to using OC, than the youngest age group. This may imply that older adults who use cannabis may continue to use opioids to address their complex health needs. Longitudinal studies that track opioid use among older adults who use cannabis will be helpful for understanding their substance use patterns and resultant health outcomes.

### 4.6 Group differences by metropolitan status

The probabilities of using a single substance versus OC also varied by type of metropolitan statistical area. Among residents of large-metro areas, the risk of using OC was higher, relative to the risk of using cannabis only, than for residents of non-metro areas. On the other hand, large-metro residents had a higher probability of using opioids only than of using OC, compared to non-metro residents. These findings may point to different pathways into substance use. In non-metro areas, where healthcare access is more limited and cannabis availability is lower (55, 56), individuals who use cannabis may be less likely to combine it with prescription opioids than those in large-metro areas. On the other hand, in large-metro areas, individuals who already use opioids may have easier access to prescription opioids and thus be less likely to complement their use with cannabis than those in non-metro areas. To better understand these pathways, more detailed longitudinal information on substance use experiences will be needed. In addition, these patterns may change if analyses incorporate both licit and illicit opioids, given geographical differences in types of opioid use (57, 58).

### 4.7 Public health implications

The findings on the characteristics of individuals who use opioids and/or cannabis have important implications for clinical care and public health. A deeper understanding of the traits associated with opioid and cannabis use or co-use can help healthcare professionals make more informed decisions about pain management, medication counseling, and follow-up care. Insights from this study may also support the development of predictive model that flag patterns of co-use across specific demographic and clinical profiles. When paired with data on other substance use behaviors and health outcomes, such tools can help identify individuals who may benefit from additional clinical attention, supportive services, or discussions about alternative therapies.

The observed variability in substance use patterns across large-metro and non-metro areas highlights the importance of geographically tailored public health efforts. In metropolitan areas, where co-use of opioids and cannabis is more common, public health campaigns that address the risks of concurrent use, particularly in regions where both substances are legally accessible, can help raise awareness and support informed decision-making. In addition, incorporating enhanced screening and patient-provider communication about polysubstance use during routine visits, especially in pain management and behavioral health settings, may help mitigate potential adverse outcomes. In non-metropolitan areas, expanding access to telemedicine for pain management and behavioral health can improve understanding the reasons individuals use opioids or cannabis and play a preventive role by offering earlier support and alternatives before other substances are introduced. Furthermore, community-based outreach programs can provide substance use education and stigma reduction tailored to rural populations, which may help reduce the risk of co-use and related harms.

Across regions, enhanced monitoring of polysubstance use, involving opioids and cannabis, can help detect early signals of emerging public health concerns and better identify populations at elevated risk. Building on the current study with temporal–spatial analyses of co-use patterns and associated health outcomes could further inform the geographic distribution of need. Such analyses can guide the strategic deployment of prevention and education initiatives, as well as the implementation of integrated care models in areas where co-use is more prevalent. This approach may support early intervention and promote safer, more coordinated management of opioid and cannabis use.

### 4.8 Limitations

Our study has several limitations. First, the NSDUH survey data are based on self-reporting of drug use and health status, and our results may be subject to recall bias influenced by social desirability (59). Second, the survey asked about general cannabis use without details about illegal use or purposes of use (i.e., medical, leisure, or both). Substance use behaviors and risks would be different among those who use cannabis for medical purposes only and among those who use it for leisure purposes only. More detailed information about cannabis use would elucidate its increasing use trends and identify individuals at a high risk of misusing it, either by itself or with other substances. Lastly, since the survey does not include drug use among those who are institutionalized, homeless, or in the military, our study results are limited to U. S. civilian, non-institutionalized individuals.

## 5 Conclusion

Individuals who use opioids only, cannabis only, or both have different demographic and health-related characteristics. Recognizing and understanding these differences is crucial for developing targeted prevention and intervention strategies that can effectively reduce the risk of co-use and mitigate its associated negative outcomes.

## Data Availability

Publicly available datasets were analyzed in this study. This data can be found at: https://www.samhsa.gov/data/data-we-collect/nsduh-national-survey-drug-use-and-health.
